# Supplementary material for: Experimental evidence that changing beliefs about mask efficacy and social norms increase mask wearing for COVID-19 risk reduction: Results from the United States and Italy
Source: PLoS One. 2021 Oct 11;16(10):e0258282. doi: 10.1371/journal.pone.0258282 (PMC8504748; doi:10.1371/journal.pone.0258282)
Supplement: S2 Appendix — (DOCX) [file pone.0258282.s002.docx]

**S2 Progression of the Experiment and Randomizations**

Participants Recruited

Consent

Demographics and covariates

Between-subjects mask efficacy randomization

Masks Protect You

Placebo Control

Masks Protect You

Scenarios below appear in a randomized order

OWN scenario

- (ATM, PARK or MEETING)

- (Others wearing or not)

OTHERS scenario

- (ATM, PARK or MEETING)

- (Others wearing or not)

THIRD PARTY scenario

- (ATM, PARK or MEETING)

- (Others wearing or not)

Sample Sizes by Treatment

| Treatment | U.S. Sample Size | Italian Sample Size |
| --- | --- | --- |
| **Mask Efficacy Treatments** |  |  |
| Untreated Control | 965 | 855 |
| Masks Protect You | 1,023 | 882 |
| Masks Protect Others | 1,112 | 850 |
| **Vignette Treatments** |  |  |
| ATM Scenario |  |  |
| OWN | 1,011 | 856 |
| OTHERS | 1,067 | 867 |
| THIRD PARTY | 1,022 | 864 |
| Park Scenario |  |  |
| OWN | 985 | 868 |
| OTHERS | 1,055 | 870 |
| THIRD PARTY | 1,060 | 849 |
| Meeting Scenario |  |  |
| OWN | 1,104 | 863 |
| OTHERS | 978 | 850 |
| THIRD PARTY | 1,018 | 874 |
| **Behavior of Others** |  |  |
| ATM Scenario |  |  |
| Both people in line are wearing their masks properly | 1,559 | 1,274 |
| Neither person in line is wearing their mask properly | 1,541 | 1,313 |
| Park Scenario |  |  |
| Almost all of the other people at the park are properly wearing a face mask | 1,528 | 1,294 |
| Not many of the other people at the park are properly wearing a face mask | 1,572 | 1,293 |
| Meeting Scenario |  |  |
| All of the other guests are properly wearing a face mask | 1,555 | 1,279 |
| None of the other guests are properly  wearing a face mask | 1,545 | 1,308 |
