# Supplementary material for: Experimental evidence that changing beliefs about mask efficacy and social norms increase mask wearing for COVID-19 risk reduction: Results from the United States and Italy
Source: PLoS One. 2021 Oct 11;16(10):e0258282. doi: 10.1371/journal.pone.0258282 (PMC8504748; doi:10.1371/journal.pone.0258282)
Supplement: S3 Appendix — (DOCX) [file pone.0258282.s003.docx]

S3 Covariates and Text of Pre-Treatment Mask Wearing Items

**United States Covariates**

*Age* in years

*Gender*, entered as categories (Male, Female, Other/Missing)

*Household Income* (Categories provided by Lucid, with a separate indicator for choosing not to supply income/missing)

*Education*, entered as categories (As provided by Lucid)

*Partisanship* (Indicators for each of the 7 standard partisanship categories, Strong Republican, Weak Republican, Lean Republican, Independent/Other/Missing, Lean Democrat, Weak Democrat, Strong Democrat)

*Race* (Indicators for Black, Latino, and Other Races)

*Employment* (Working at home, working outside home by choice, Working outside home because required, not working)

*Flu shot* In the last 5 years, how many times have you gotten the seasonal flu vaccine (flu shot)? (0, 1, 2, 3, 4, 5)

**Italy Covariates**

*Age* in years

*Gender*, entered as categories (Male, Female, Other/Missing)

*Household Income* (Categories provided by Lucid, with a separate indicator for choosing not to supply income/missing)

*Education*, entered as categories (As provided by Lucid)

*Vote (*Indicator for each of 7 alternatives regarding vote in 2016 elections: Did not vote, Potere al Popolo, Liberi e Uguali, Partito Democratico (coalition), Forza Italia/Lega/Fratelli D’Italia, Casa Pound, Movimento 5 Stelle, Doesn’t know).

*Employment* (Working at home, working outside home by choice, Working outside home because required, not working)

*Flu shot* In the last 5 years, how many times have you gotten the seasonal flu vaccine (flu shot)? (0, 1, 2, 3, 4, 5)

**Mask Wearing Items**

*Your Mask Use*

How often do you wear a mask?

- I refuse to wear a mask, even when it is required by a business or law.
- I only wear a mask when I’m required to by a business or law.
- I occasionally wear a mask when I’m out in public, even if it is not required.
- I usually wear a mask when I’m out in public, even if it is not required.
- I always wear a mask when I’m out in public, even if it is not required.

*Others Mask Use*

When you go out in your neighborhood and community, how many people that you see out in public are wearing a mask?

- 0-20% (1 out of 5 or fewer people)
- 21-40% (between 1 out of 5 and 2 out of 5 people)
- 41-60% (about half)
- 61-80% (between 3 out of 5 and 4 out of 5 people)
- 81-100% (more than 4 out of 5 people)

**Table: Analysis for Figure 1**

|  | United States | | | |  | Italy | | | |
| --- | --- | --- | --- | --- | --- | --- | --- | --- | --- |
|  | (1) | (2) | (3) | (4) |  | (5) | (6) | (7) | (8) |
|  | Agree masks protect others | Strongly Agree masks protect others (binary) | Agree masks protect you | Strongly Agree masks protect you (binary) |  | Agree masks protect others | Strongly Agree masks protect others (binary) | Agree masks protect you | Strongly Agree masks protect you (binary) |
| T: Masks protect you | -0.005 | 0.003 | 0.061 | 0.085 |  | -0.003 | -0.024 | 0.06 | 0.062 |
|  | [0.012] | [0.022] | [0.012]*** | [0.022]*** |  | [0.011] | [0.024] | [0.013]*** | [0.023]*** |
| T: Masks protect others | 0.031 | 0.068 | 0.057 | 0.098 |  | 0.017 | 0.04 | 0.064 | 0.098 |
|  | [0.011]*** | [0.022]*** | [0.012]*** | [0.022]*** |  | [0.011] | [0.024]* | [0.013]*** | [0.024]*** |
| Observations | 2905 | 2905 | 2904 | 2904 |  | 2549 | 2549 | 2550 | 2550 |
| R-squared | 0.070 | 0.067 | 0.095 | 0.090 |  | 0.049 | 0.036 | 0.044 | 0.026 |
| Mean of DV | 0.813 | 0.545 | 0.784 | 0.497 |  | 0.827 | 0.507 | 0.772 | 0.416 |
| S.D. of DV | 0.260 | 0.498 | 0.277 | 0.500 |  | 0.224 | 0.5 | 0.258 | 0.493 |

Corresponding OLS coefficients with robust standard errors in brackets for Figure 1. * significant at 10%; ** significant at 5%; *** significant at 1%. Placebo control condition of the mask efficacy treatment is the omitted category. Control variables and constant omitted from regression plots. Covariates are age (years), gender, household income, ethnicity (White, Black, Asian, Other), education, partisanship, work status, and previous flu vaccination. See Supplemental Information S-2 for control variable coding.

**Table: Analysis for Figure 2 Panel A and Panel B**

|  | United States | | |  | Italy | | |
| --- | --- | --- | --- | --- | --- | --- | --- |
|  | (1) | (2) | (3) |  | (4) | (5) | (6) |
|  | Pooled OWN scenario outcome | Pooled OWN behavior outcome, binary | Pooled OWN behavior outcome, alternative coding (leave or get mask), binary |  | Pooled OWN scenario outcome | Pooled OWN behavior outcome, binary | Pooled OWN behavior outcome, alternative coding (leave or get mask), binary |
| T: Masks protect you | 0.027 | 0.005 | 0.016 |  | -0.031 | -0.009 | -0.013 |
|  | [0.048] | [0.022] | [0.021] |  | [0.040] | [0.022] | [-0.018] |
| T: Masks protect others | 0.139 | 0.057 | 0.058 |  | -0.052 | -0.027 | -0.016 |
|  | [0.047]*** | [0.021]*** | [0.020]*** |  | [0.040] | [0.022] | [0.018] |
| T: Others wearing masks | 0.295 | 0.173 | 0.084 |  | 0.233 | 0.163 | 0.057 |
|  | [0.038]*** | [0.017]*** | [0.016]*** |  | [0.033]*** | [0.018]*** | [0.015]*** |
| Scenario is ATM | -0.462 | -0.222 | -0.228 |  | -0.3 | -0.153 | -0.148 |
|  | [0.049]*** | [0.022]*** | [0.021]*** |  | [0.042]*** | [0.022]*** | [0.019]*** |
| Scenario is MEETING | 0.077 | -0.004 | 0.058 |  | -0.062 | -0.054 | -0.005 |
|  | [0.046] | [0.021] | [0.019]*** |  | [0.038]* | [0.021]*** | [0.016] |
| Observations | 2872 | 2872 | 2872 |  | 2530 | 2530 | 2530 |
| R-squared | 0.150 | 0.130 | 0.159 |  | 0.059 | 0.061 | 0.059 |
| Mean of DV | 3.111 | 0.563 | 0.656 |  | 3.493 | 0.694 | 0.828 |
| S.D. of DV | 1.104 | 0.496 | 0.475 |  | 0.842 | 0.461 | 0.377 |

Corresponding OLS coefficients with robust standard errors in brackets for Figure 2 OWN behavior scenarios. * significant at 10%; ** significant at 5%; *** significant at 1%. Placebo control, no one/few people are wearing a mask, and the PARK scenario are the omitted categories. Control variables and constant omitted from regression plots. Covariates are age (years), gender, household income, ethnicity (White, Black, Asian, Other), education, partisanship, work status, and previous flu vaccination. See Supplemental Information S-2 for control variable coding.

**Table: Analysis for Figure 2: Panel C and Panel D**

|  | United States | |  | Italy | |
| --- | --- | --- | --- | --- | --- |
|  | (1) | (2) |  | (3) | (4) |
|  | Pooled OTHERS scenario outcome | Pooled OTHERS scenario outcome, binary |  | Pooled OTHERS scenario outcome | Pooled OTHERS scenario outcome, binary |
| T: Masks protect you | 0.037 | 0.004 |  | 0.026 | 0.033 |
|  | [0.049] | [0.021] |  | [0.047] | [0.023] |
| T: Masks protect others | 0.149 | 0.045 |  | 0.027 | 0.026 |
|  | [0.047]*** | [0.021]** |  | [0.047] | [0.023] |
| T: Others wearing masks | 0.060 | 0.084 |  | 0.143 | 0.125 |
|  | [0.039] | [0.017]*** |  | [0.039]*** | [0.019]*** |
| Scenario is ATM | -0.262 | -0.212 |  | -0.433 | -0.239 |
|  | [0.047]*** | [0.021]*** |  | [0.047]*** | [0.023]*** |
| Scenario is MEETING | -0.079 | -0.105 |  | -0.054 | -0.055 |
|  | [0.049] | [0.022]*** |  | [0.047] | [0.023] |
| Observations | 2868 | 2868 |  | 2530 | 2530 |
| R-squared | 0.086 | 0.085 |  | 0.086 | 0.091 |
| Mean of DV | 2.724 | 0.343 |  | 3.176 | 0.557 |
| S.D. of DV | 1.073 | 0.475 |  | 1.002 | 0.497 |

Corresponding OLS coefficients with robust standard errors in brackets for Figure 2 OTHERS behavior scenarios. * significant at 10%; ** significant at 5%; *** significant at 1%. Placebo control, no one/few people are wearing a mask, and the PARK scenario are the omitted categories. Control variables and constant omitted from regression plots. Covariates are age (years), gender, household income, ethnicity (White, Black, Asian, Other), education, partisanship, work status, and previous flu vaccination. See Supplemental Information S-2 for control variable coding.

**Table: Analysis for Figure 3**

|  | United States | |  | Italy | |
| --- | --- | --- | --- | --- | --- |
|  | (1) | (2) |  | (3) | (4) |
|  | Negative judgment person not wearing mask correctly | Negative judgment person asking other to fix mask |  | Negative judgment person not wearing mask correctly | Negative judgment person asking other to fix mask |
| T: Masks protect you | 0.001 | -0.022 |  | -0.002 | 0.028 |
|  | [0.010] | [0.019] |  | [0.01] | [0.019] |
| T: Masks protect others | 0.001 | -0.022 |  | 0.005 | 0.005 |
|  | [0.010] | [0.019] |  | [0.01] | [0.017] |
| T: Others wearing masks | 0.015 | -0.036 |  | 0.02 | 0.007 |
|  | [0.008]* | [0.016]** |  | [0.009]** | [0.015] |
| Scenario is ATM | -0.011 | 0.046 |  | -0.057 | 0.004 |
|  | [0.010] | [0.019]** |  | [0.01]*** | [0.018] |
| Scenario is MEETING | -0.053 | 0.006 |  | -0.096 | 0.042 |
|  | [0.010]*** | [0.018] |  | [0.011]*** | [0.016]** |
| Observations | 2865 | 686 |  | 2172 | 555 |
| R-squared | 0.101 | 0.115 |  | 0.072 | 0.094 |
| Mean of DV | 0.616 | 0.259 |  | 0.806 | 0.103 |
| S.D. of DV | 0.226 | 0.208 |  | 0.205 | 0.177 |

Corresponding OLS coefficients with robust standard errors in brackets for Figure 4. * significant at 10%; ** significant at 5%; *** significant at 1%. Placebo control, no one/few people are wearing a mask, and the PARK scenario are the omitted categories. Control variables and constant omitted from regression plots. Covariates are age (years), gender, household income, ethnicity (White, Black, Asian, Other), education, partisanship, work status, and previous flu vaccination. See Supplemental Information S-2 for control variable coding.
