# Supplementary material for: Experimental evidence that changing beliefs about mask efficacy and social norms increase mask wearing for COVID-19 risk reduction: Results from the United States and Italy
Source: PLoS One. 2021 Oct 11;16(10):e0258282. doi: 10.1371/journal.pone.0258282 (PMC8504748; doi:10.1371/journal.pone.0258282)
Supplement: S4 Appendix — (DOCX) [file pone.0258282.s004.docx]

S4 First Scenario Robustness Analysis

**Figure: Own Scenario Shown First**

| United States | Italy |
| --- | --- |
| **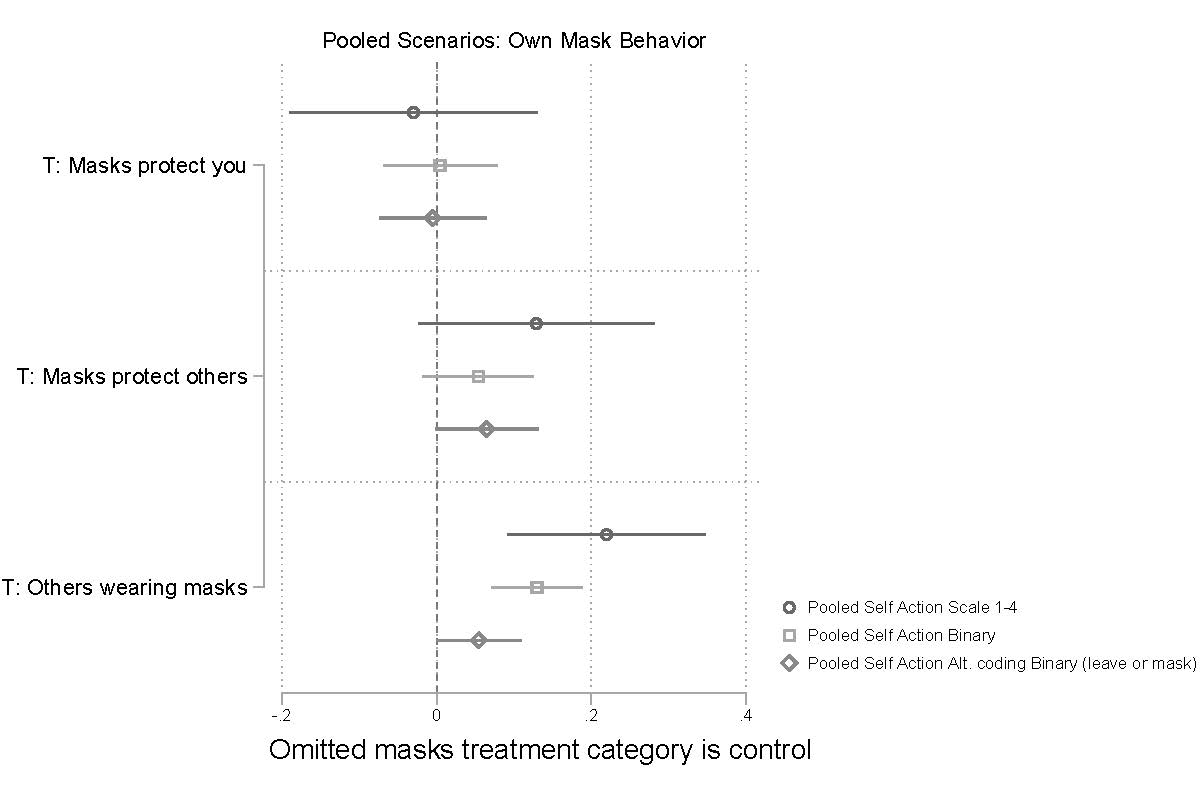** | **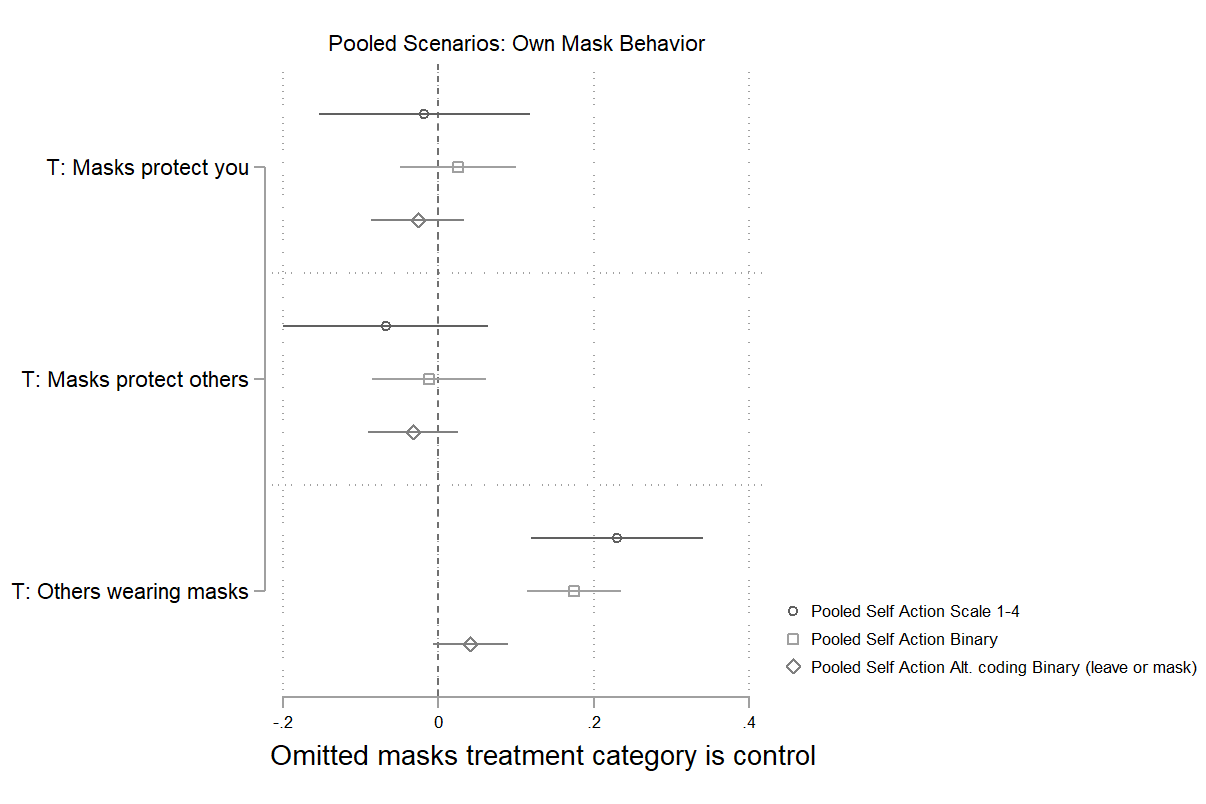** |

Effect of mask efficacy treatments and social norms treatment on reported OWN mask behavior for respondents who saw an OWN mask behavior scenario first. The figure displays OLS regression estimates with 95% confidence intervals. Models included covariates described above.

**Figure: Other Scenario Shown First**

| United States | Italy |
| --- | --- |
| **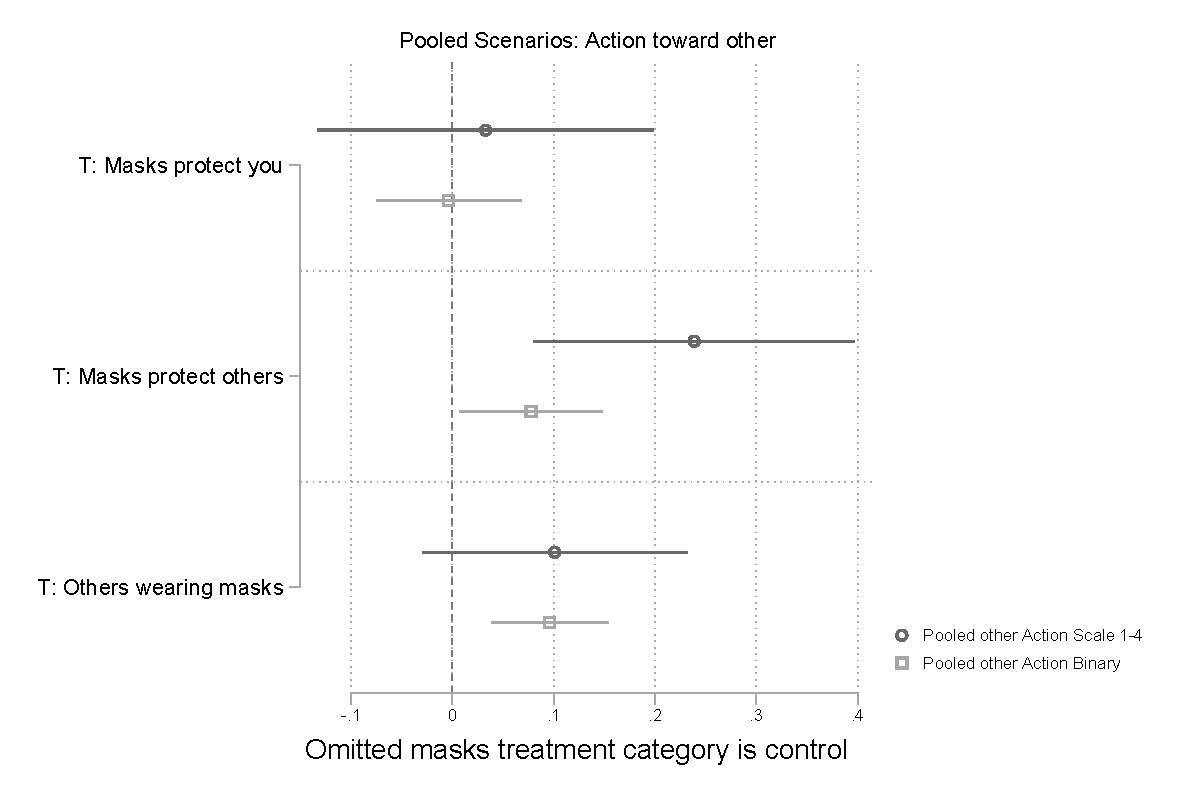** | **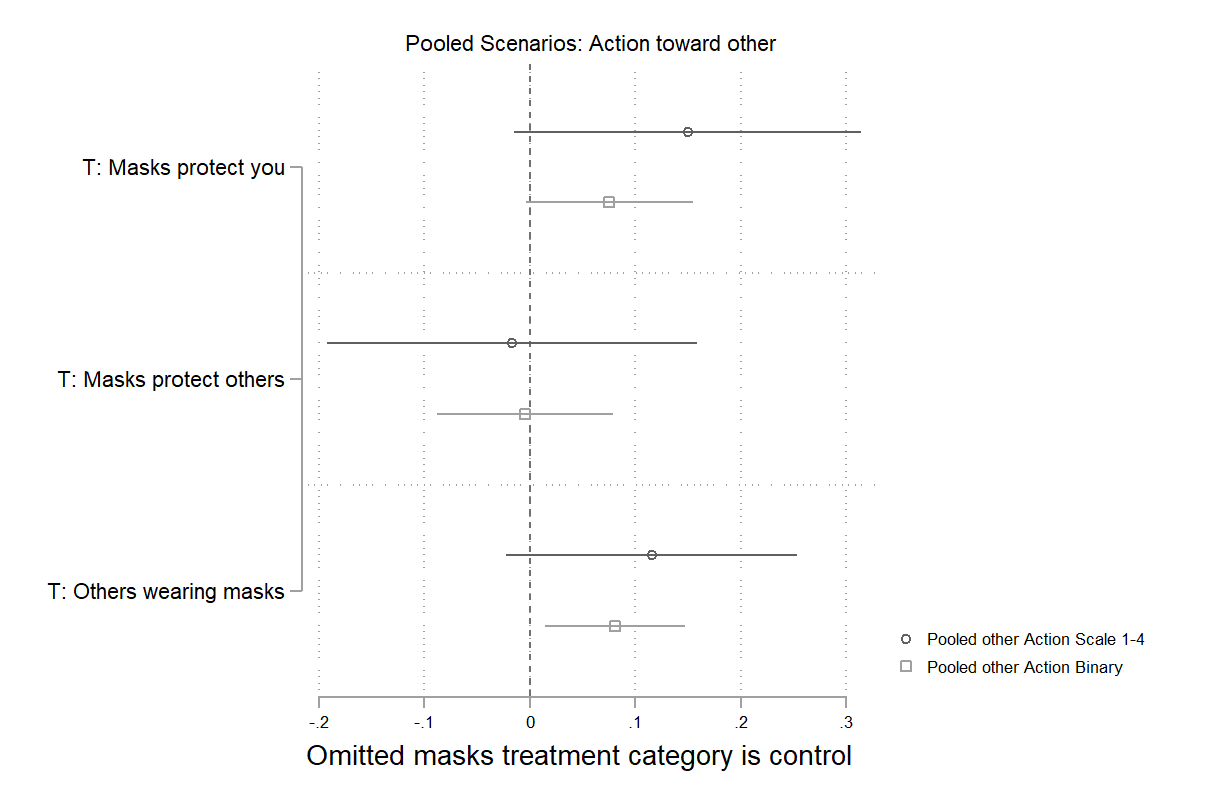** |

Effect of mask efficacy treatments and social norms treatment on reported OTHER mask behavior for respondents who saw an OTHER mask behavior scenario first. The figure displays OLS regression estimates with 95% confidence intervals. Models included covariates described above.
