# Supplementary material for: Experimental evidence that changing beliefs about mask efficacy and social norms increase mask wearing for COVID-19 risk reduction: Results from the United States and Italy
Source: PLoS One. 2021 Oct 11;16(10):e0258282. doi: 10.1371/journal.pone.0258282 (PMC8504748; doi:10.1371/journal.pone.0258282)
Supplement: S5 Appendix — (DOCX) [file pone.0258282.s005.docx]

S5 Scenario Level Analysis

**Figure: OWN behavior for ATM Scenario**

| United States | Italy |
| --- | --- |
| **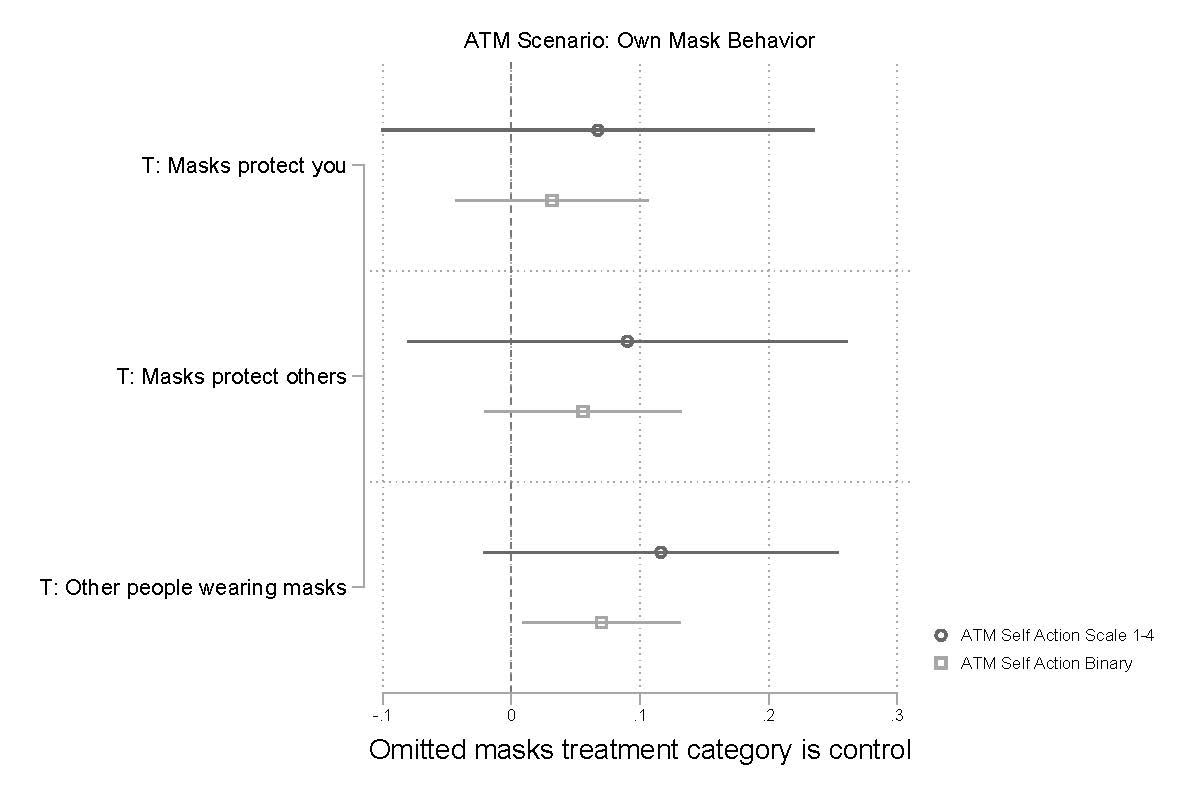** | **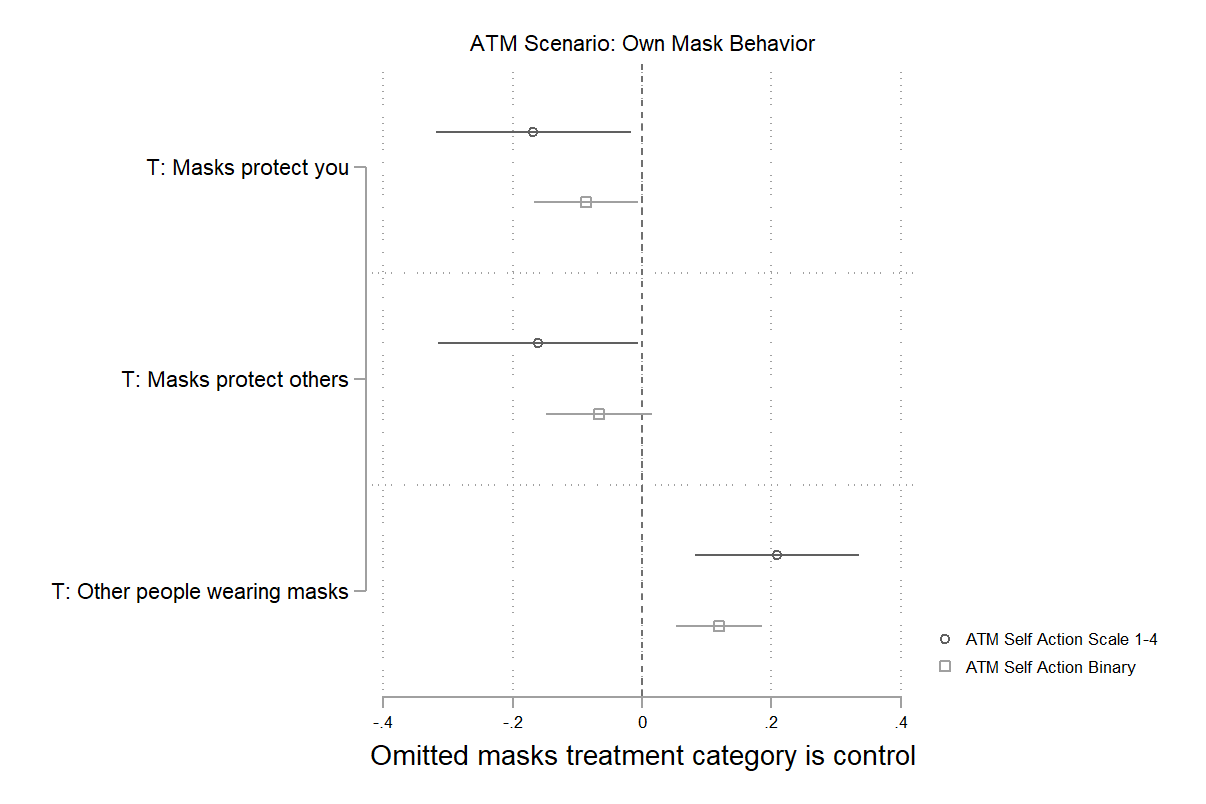** |

Effect of mask efficacy treatments and social norms treatment on reported OWN mask behavior for the ATM scenario. The figure displays OLS regression estimates with 95% confidence intervals. Models included covariates described above.

**Figure: OTHERS behavior for ATM Scenario**

| United States | Italy |
| --- | --- |
| **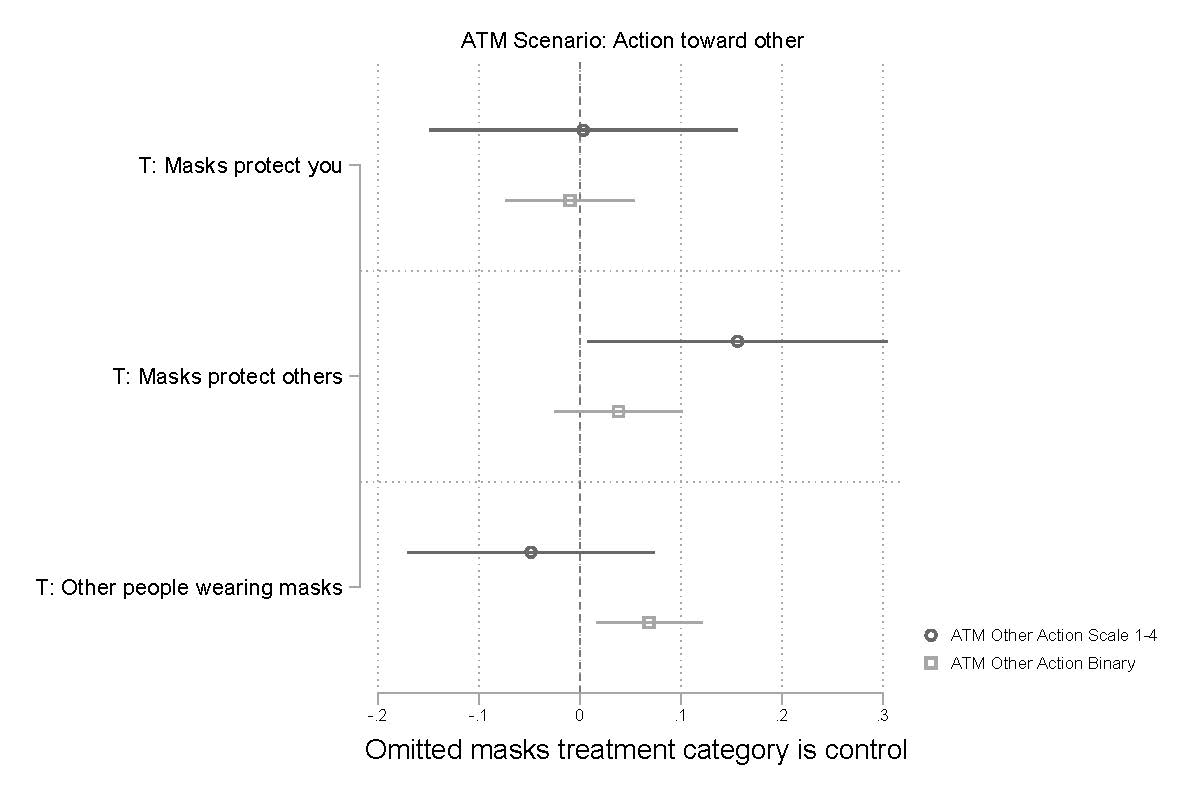** | **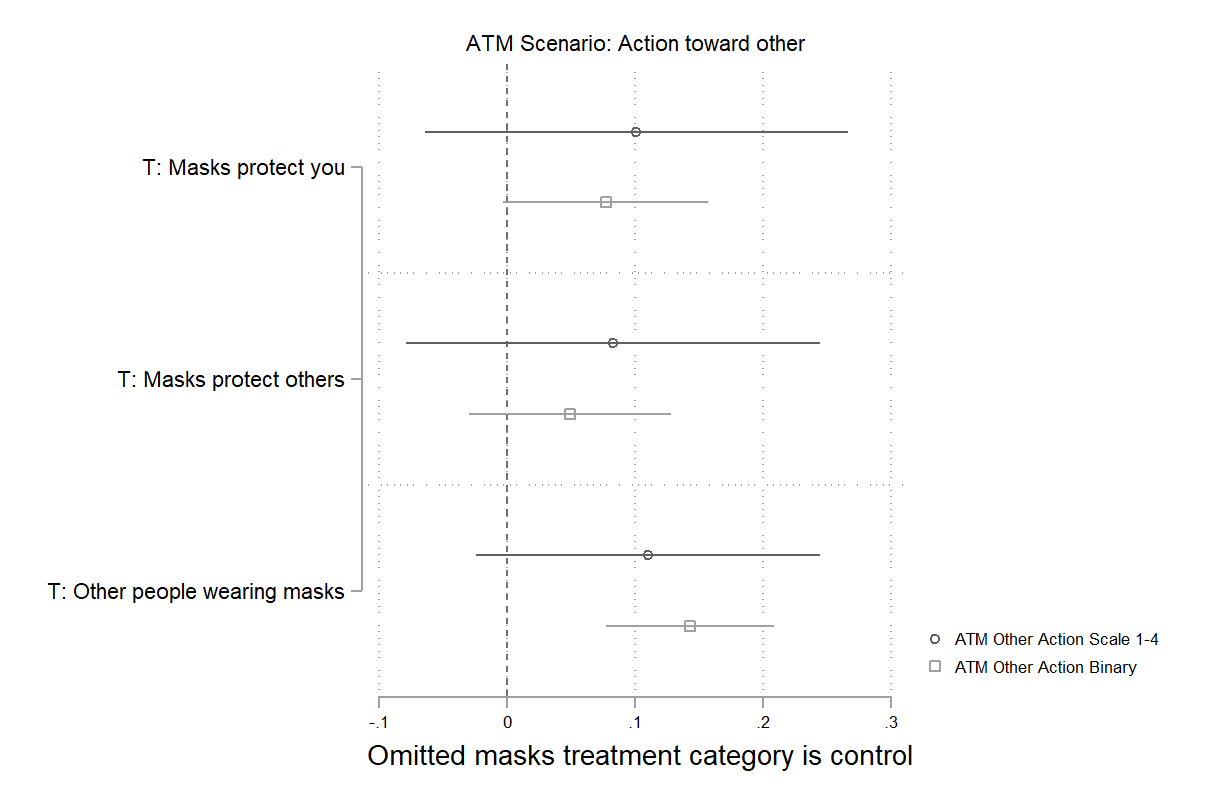** |

Effect of mask efficacy treatments and social norms treatment on reported action towards OTHERS for the ATM scenario. The figure displays OLS regression estimates with 95% confidence intervals. Models included covariates described above.

**Figure: THIRD PARTY for ATM Scenario**

| United States | Italy |
| --- | --- |
| **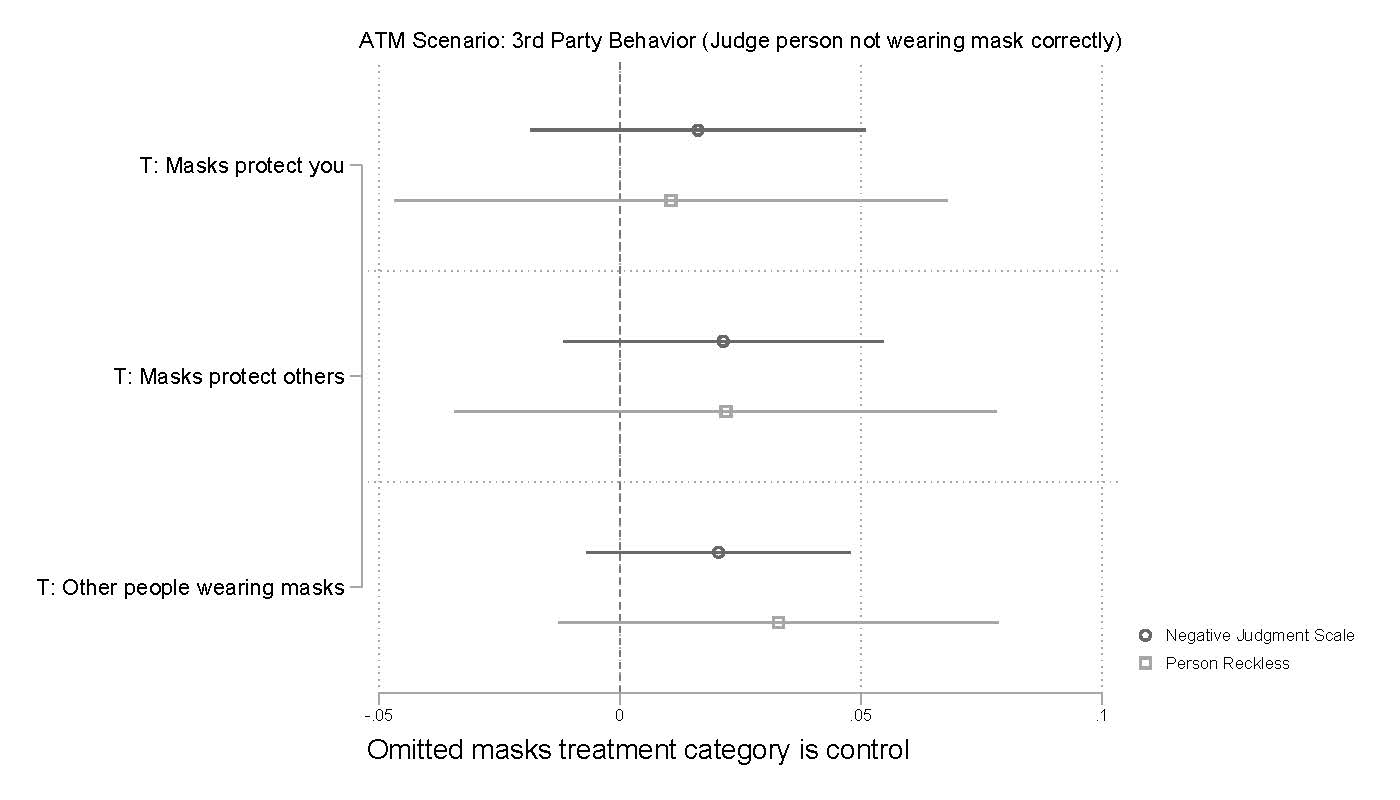** | **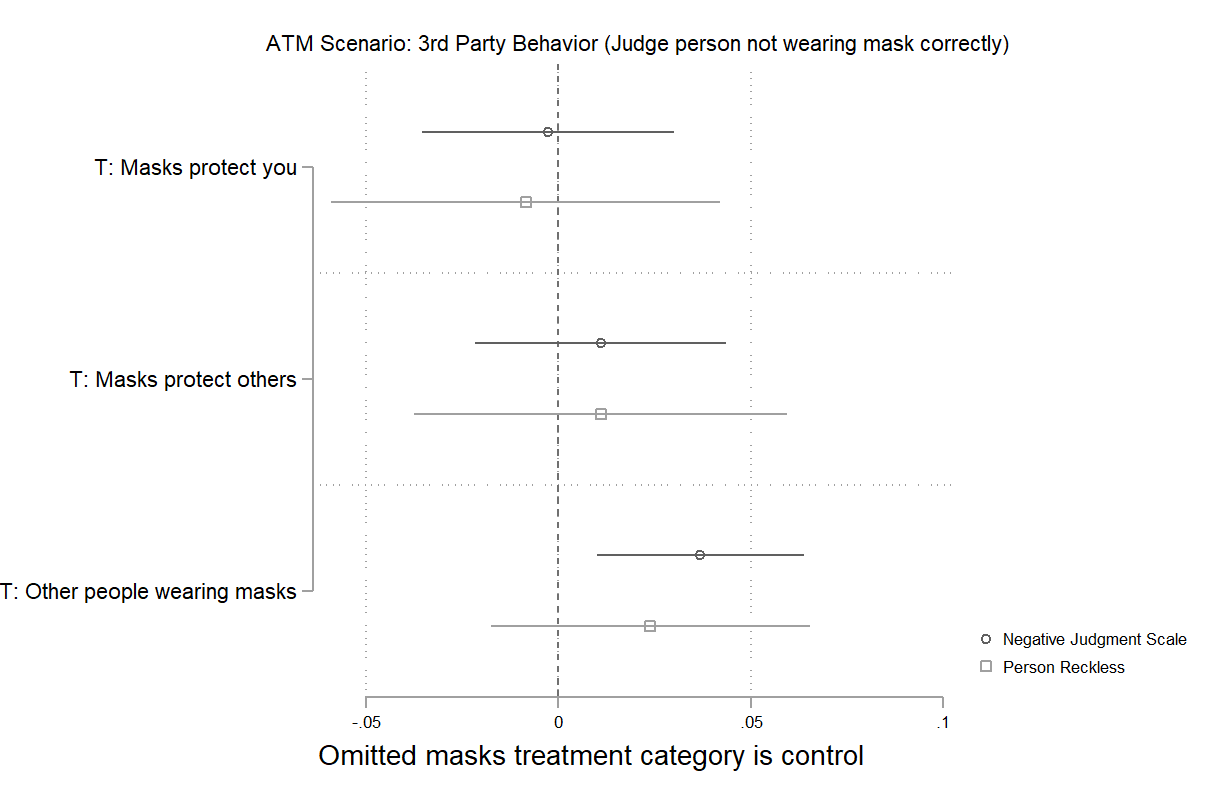** |

Effect of mask efficacy treatments and social norms treatment on reported judgment of person who is not wearing their mask correctly mask behavior for the THIRD PARTY version of ATM scenario. The figure displays OLS regression estimates with 95% confidence intervals. Models included covariates described above.

**Figure: THIRD PARTY for ATM Scenario ask to fix**

| United States | Italy |
| --- | --- |
| **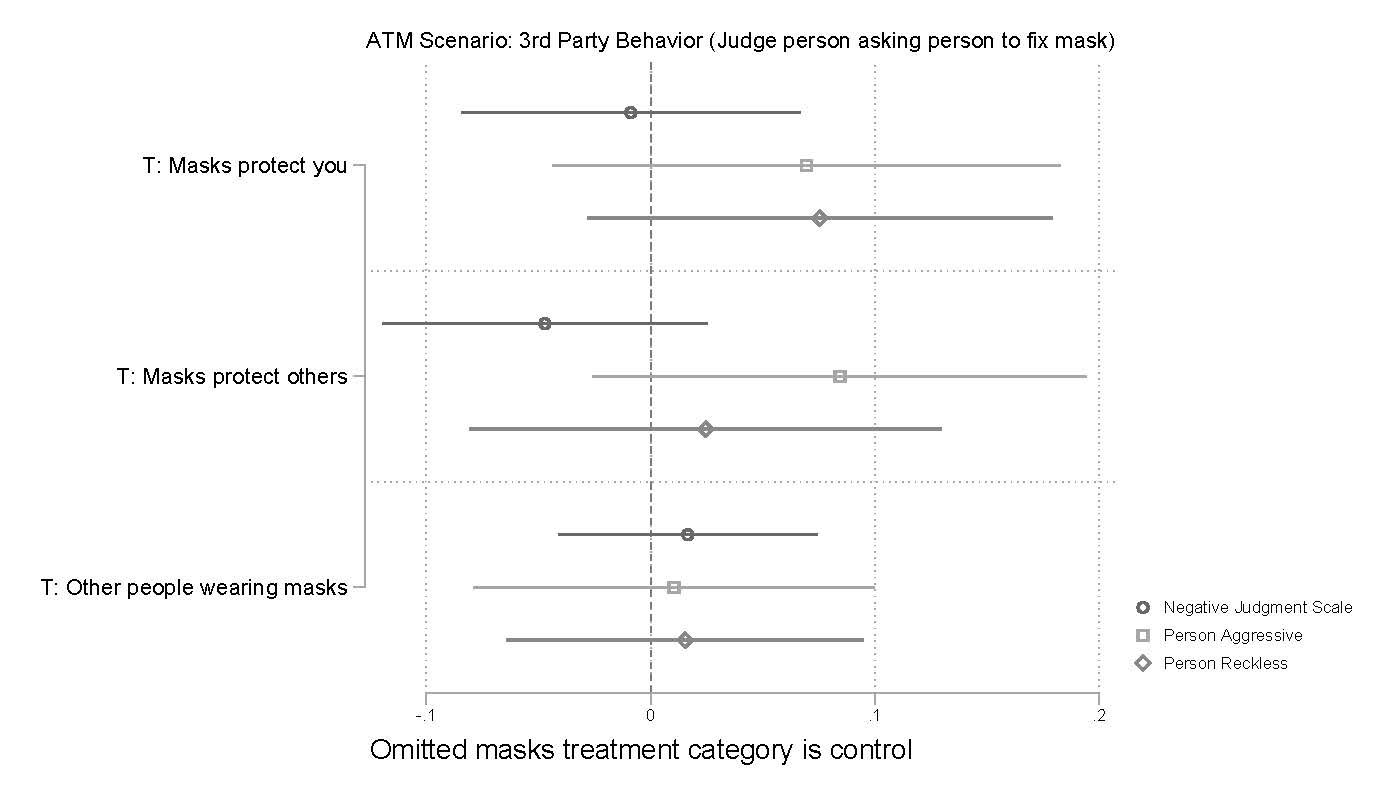** | **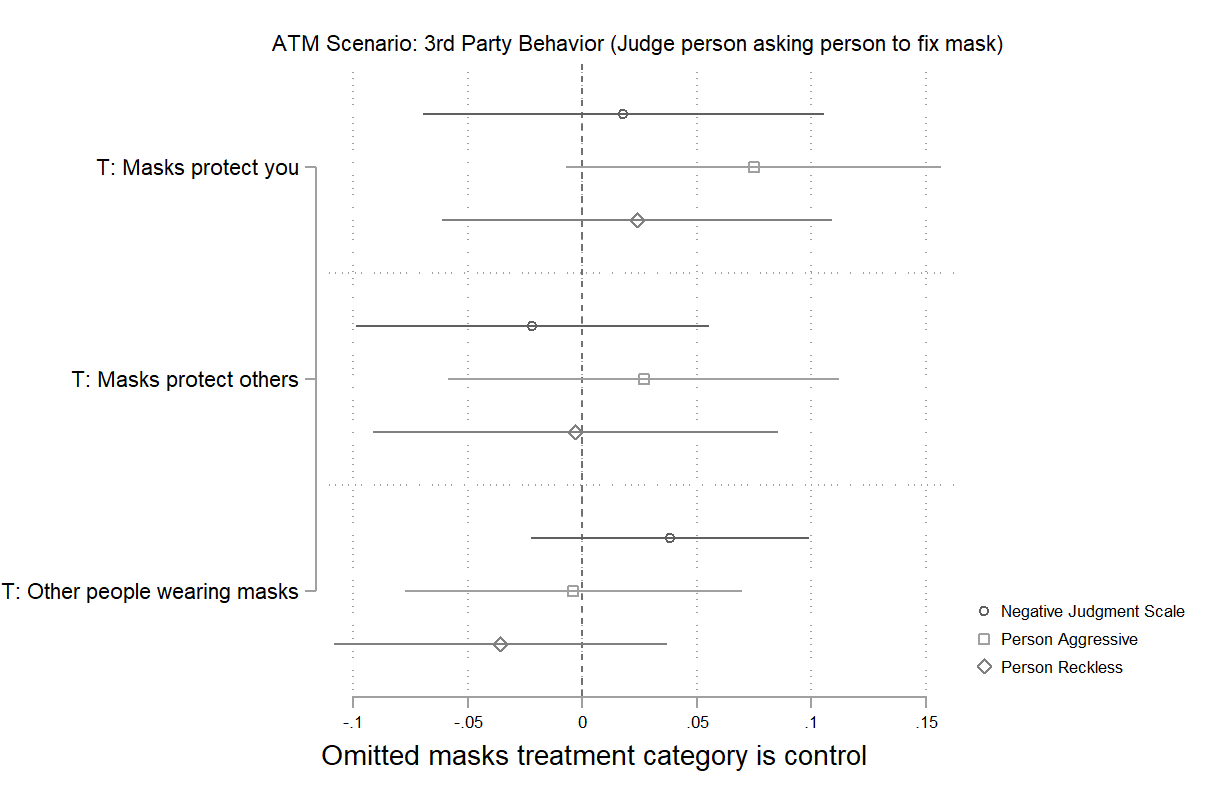** |

Effect of mask efficacy treatments and social norms treatment on reported judgment of person who asked someone to fix their mask in the THIRD PARTY version of ATM scenario. The figure displays OLS regression estimates with 95% confidence intervals. Models included covariates described above.

**Figure: OWN behavior for PARK Scenario**

| United States | Italy |
| --- | --- |
| **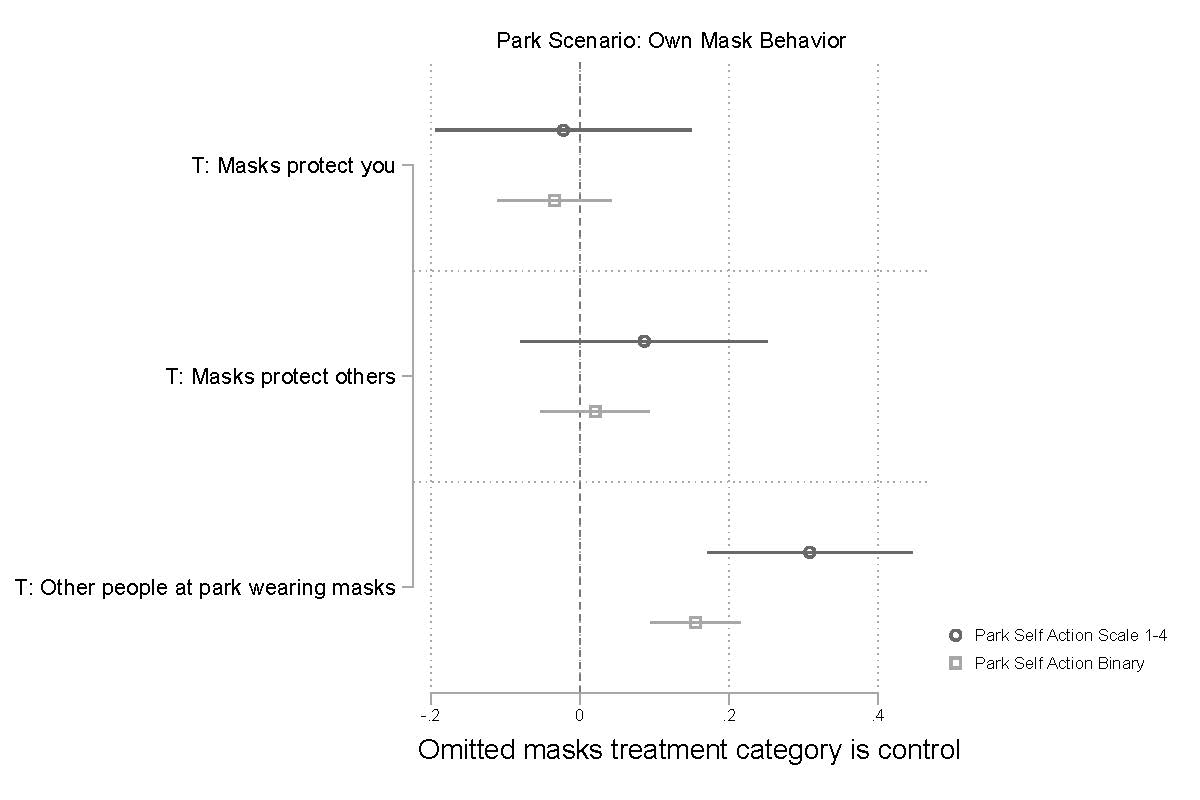** | **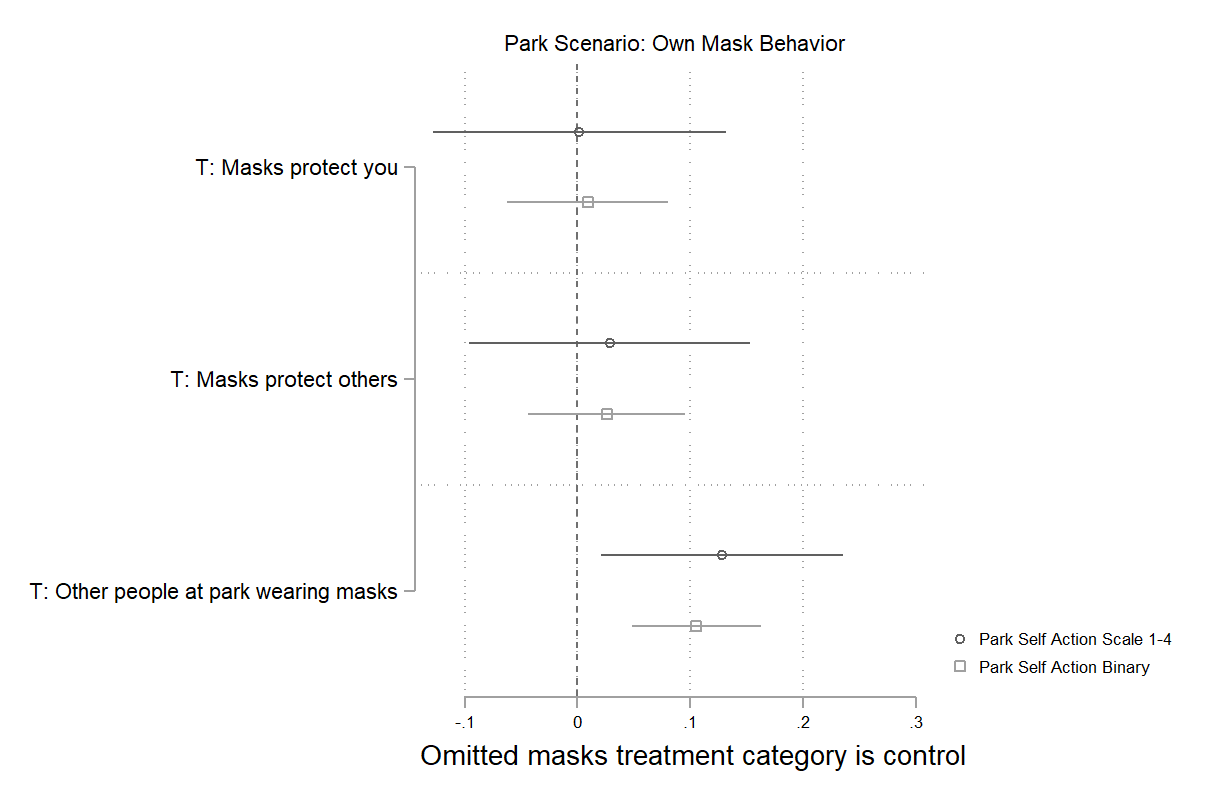** |

Effect of mask efficacy treatments and social norms treatment on reported OWN mask behavior for the PARK scenario. The figure displays OLS regression estimates with 95% confidence intervals. Models included covariates described above.

**Figure: OTHERS behavior for PARK Scenario**

| United States | Italy |
| --- | --- |
| **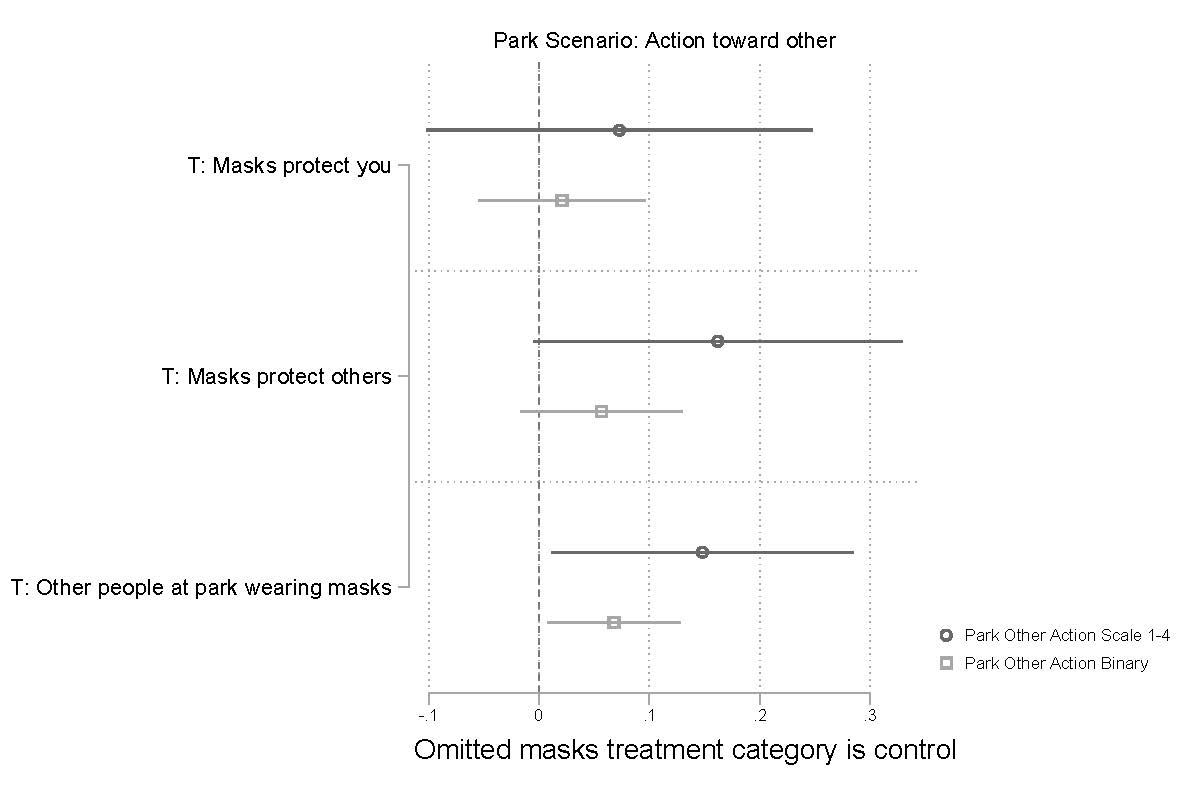** | **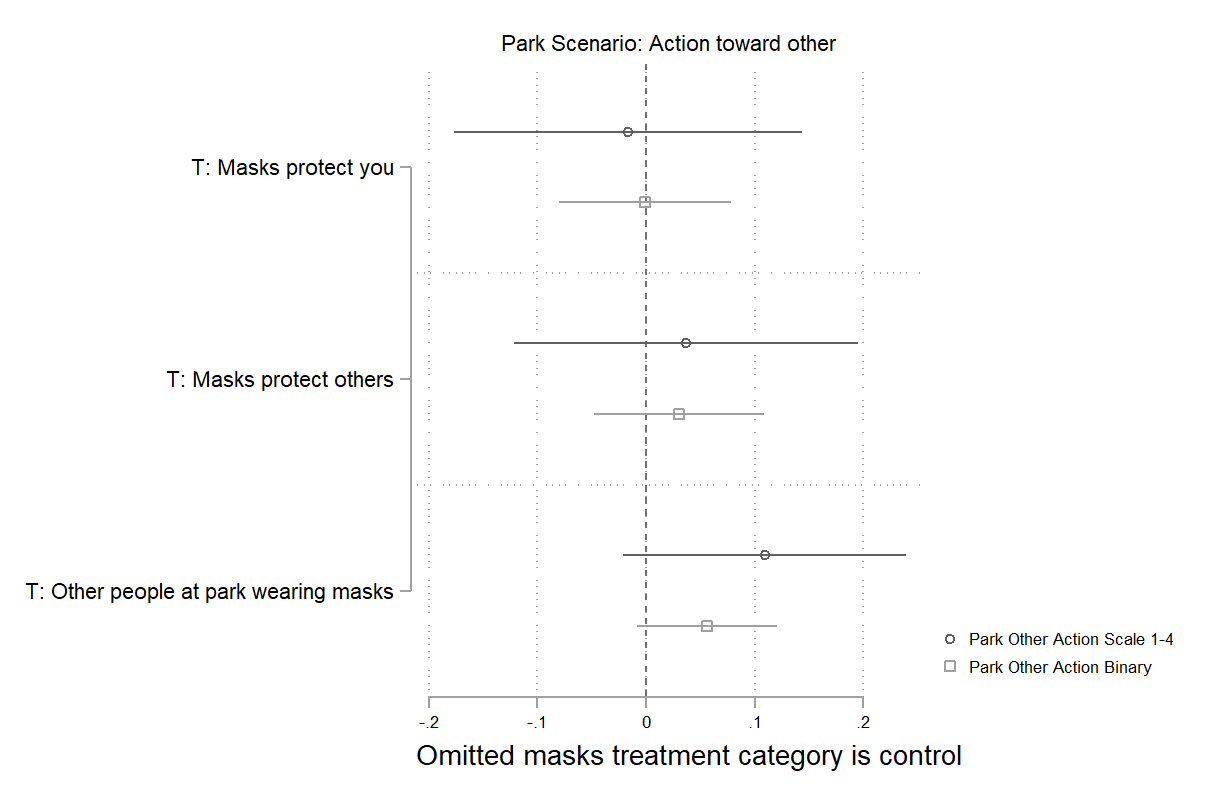** |

Effect of mask efficacy treatments and social norms treatment on reported action towards OTHERS for the PARK scenario. The figure displays OLS regression estimates with 95% confidence intervals. Models included covariates described above.

**Figure: THIRD PARTY behavior for PARK Scenario**

| United States | Italy |
| --- | --- |
| **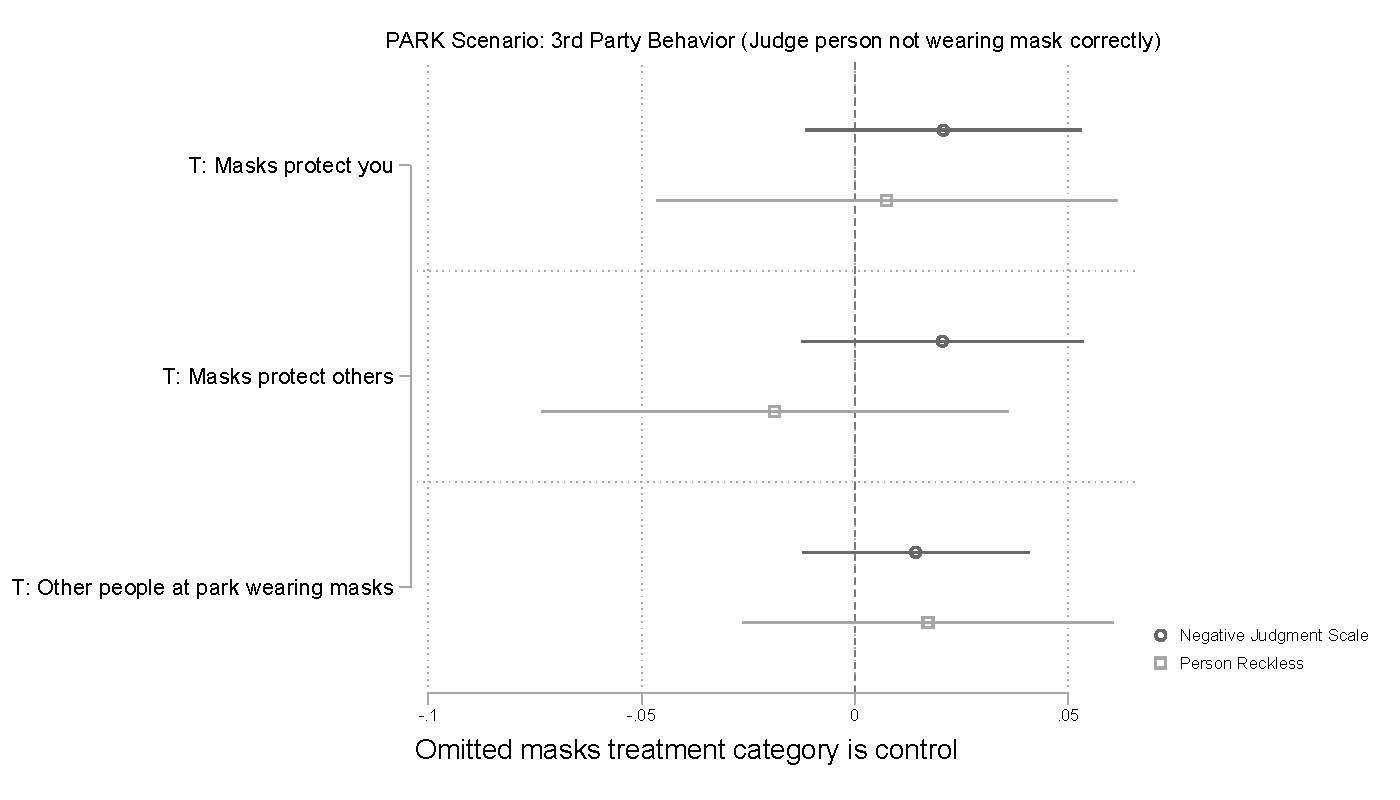** | **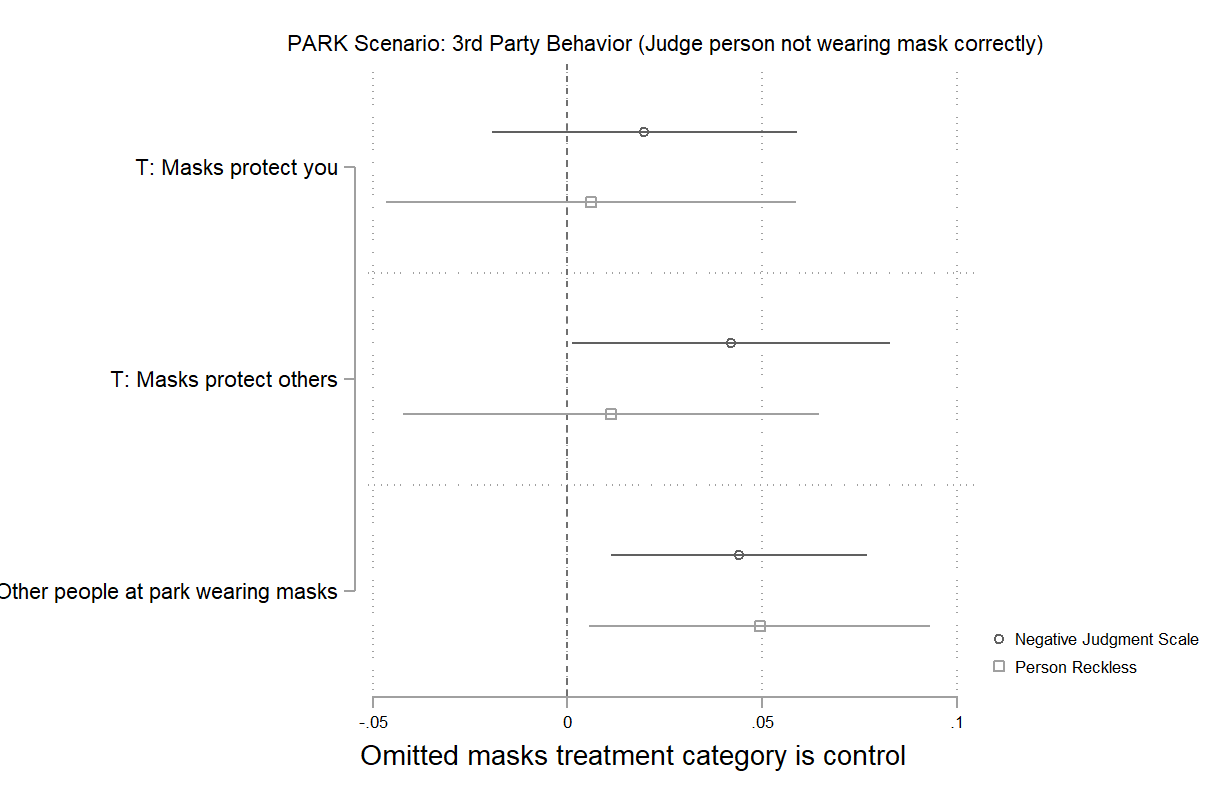** |

Effect of mask efficacy treatments and social norms treatment on reported judgment of person who is not wearing their mask correctly mask behavior for the THIRD PARTY version of PARK scenario. The figure displays OLS regression estimates with 95% confidence intervals. Models included covariates described above.

**Figure: THIRD PARTY behavior for PARK Scenario ask to fix**

| United States | Italy |
| --- | --- |
| **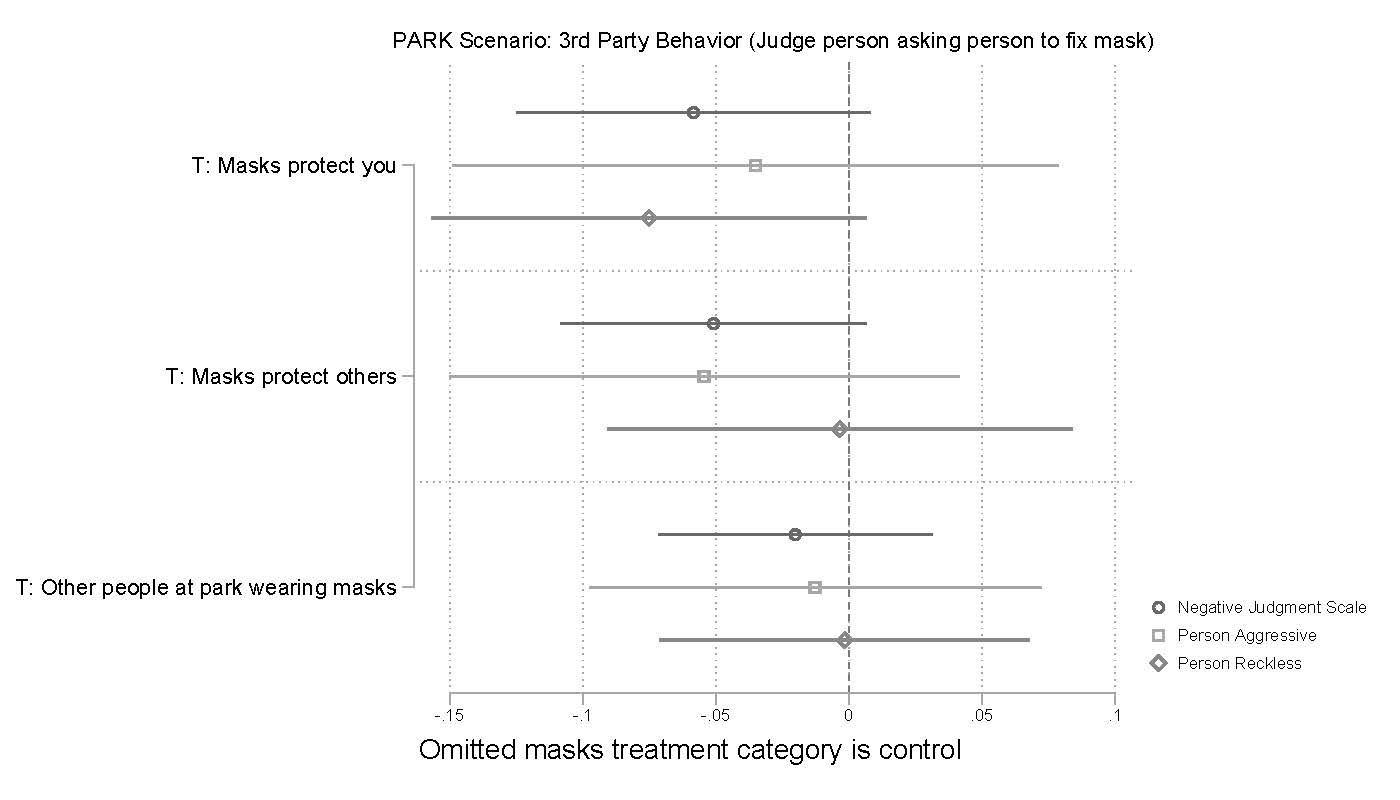** | **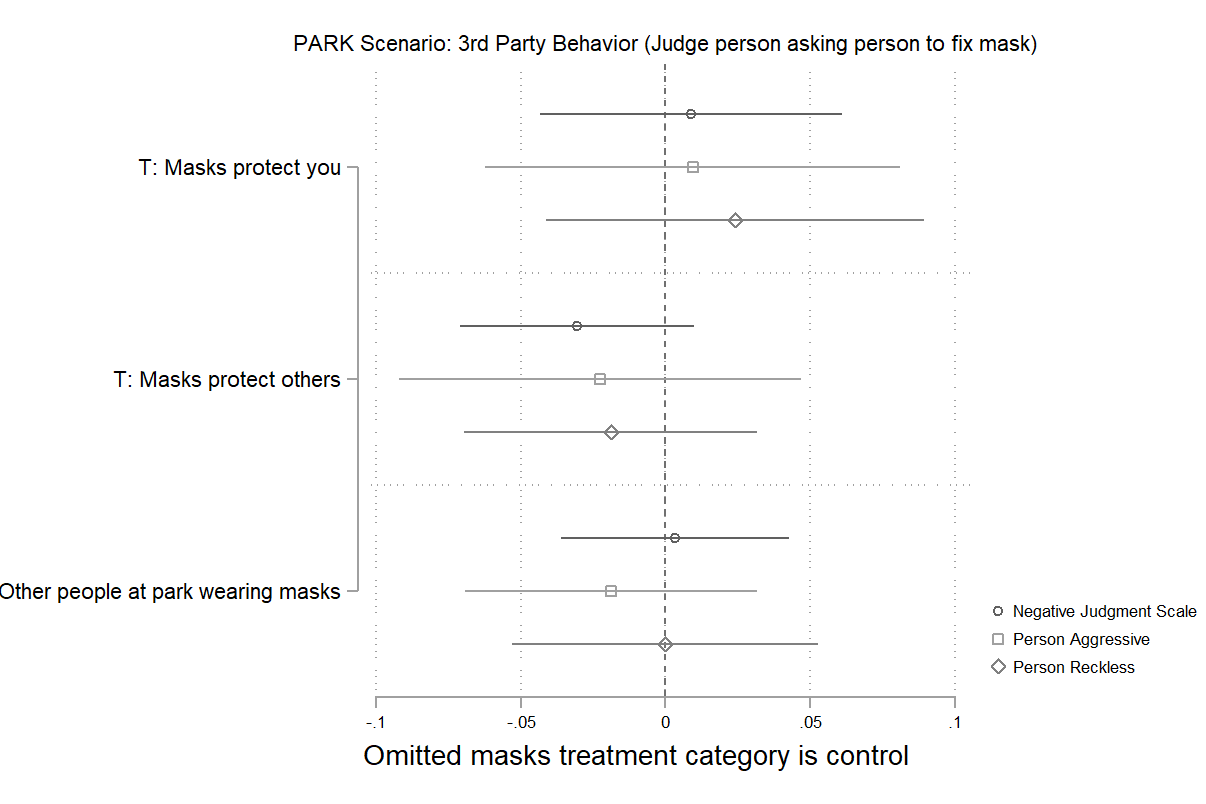** |

Effect of mask efficacy treatments and social norms treatment on reported judgment of person who asked someone to fix their mask in the THIRD PARTY version of PARK scenario. The figure displays OLS regression estimates with 95% confidence intervals. Models included covariates described above.

**Figure: OWN behavior for MEETING Scenario**

| United States | Italy |
| --- | --- |
| **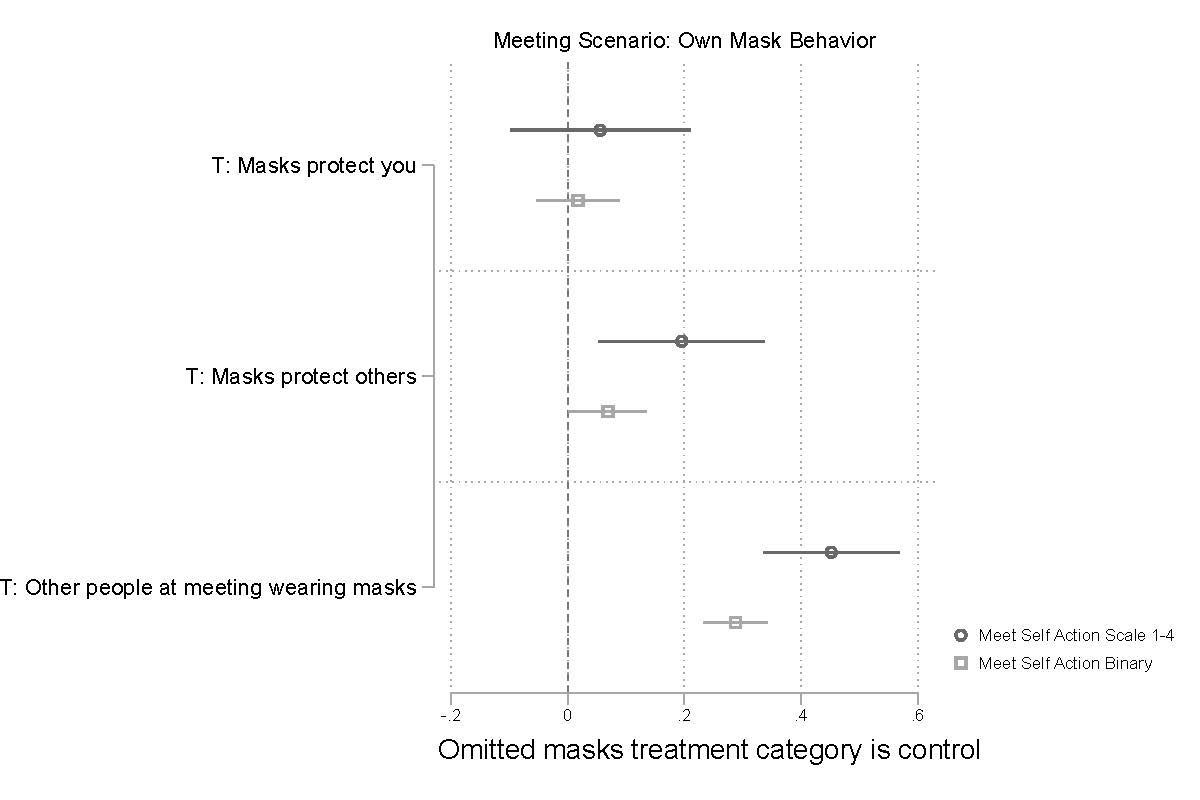** | **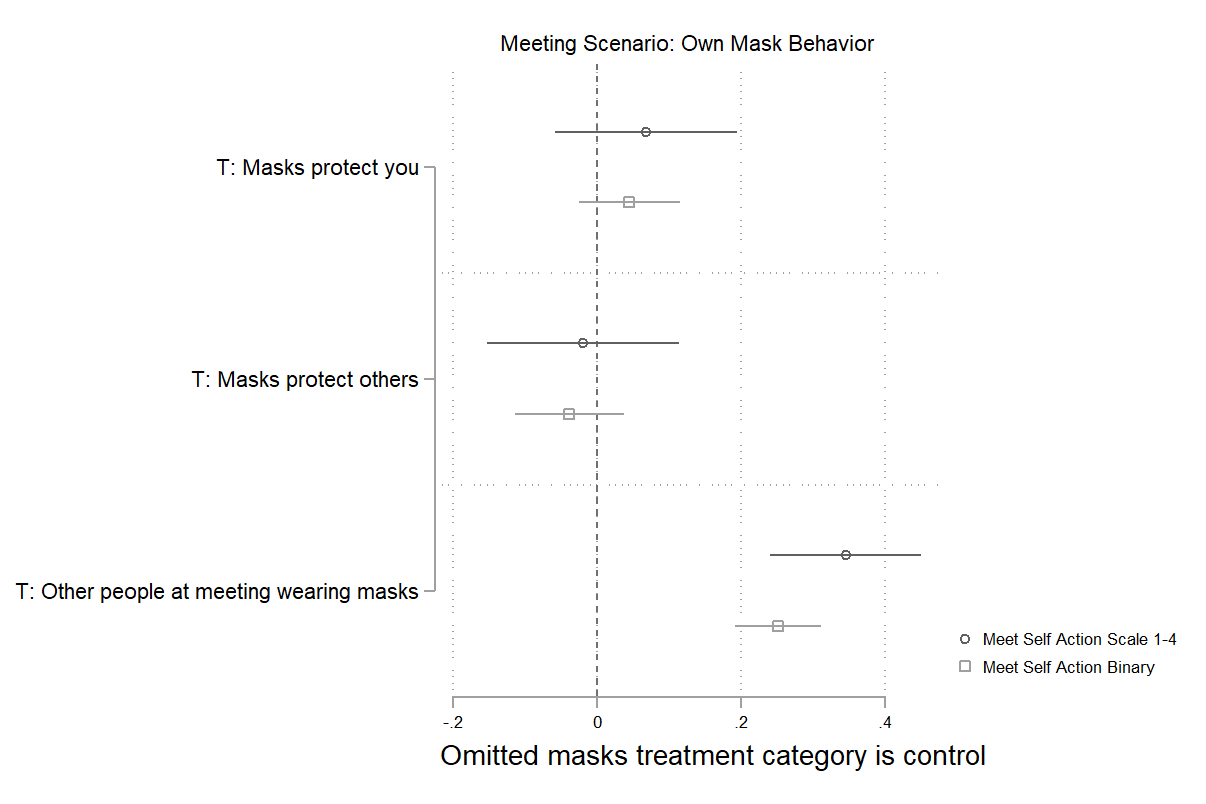** |

Effect of mask efficacy treatments and social norms treatment on reported OWN mask behavior for the MEETING scenario. The figure displays OLS regression estimates with 95% confidence intervals. Models included covariates described above.

**Figure: OTHERS behavior for MEETING Scenario**

| United States | Italy |
| --- | --- |
| **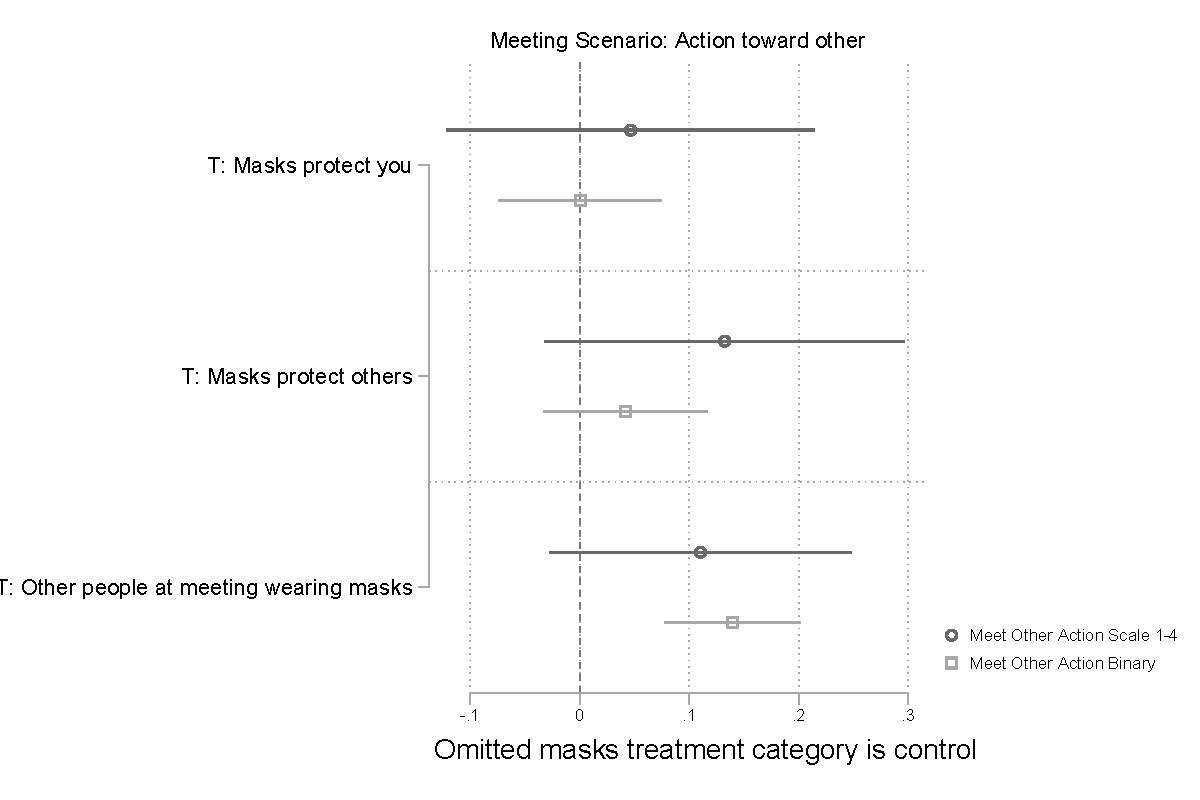** | **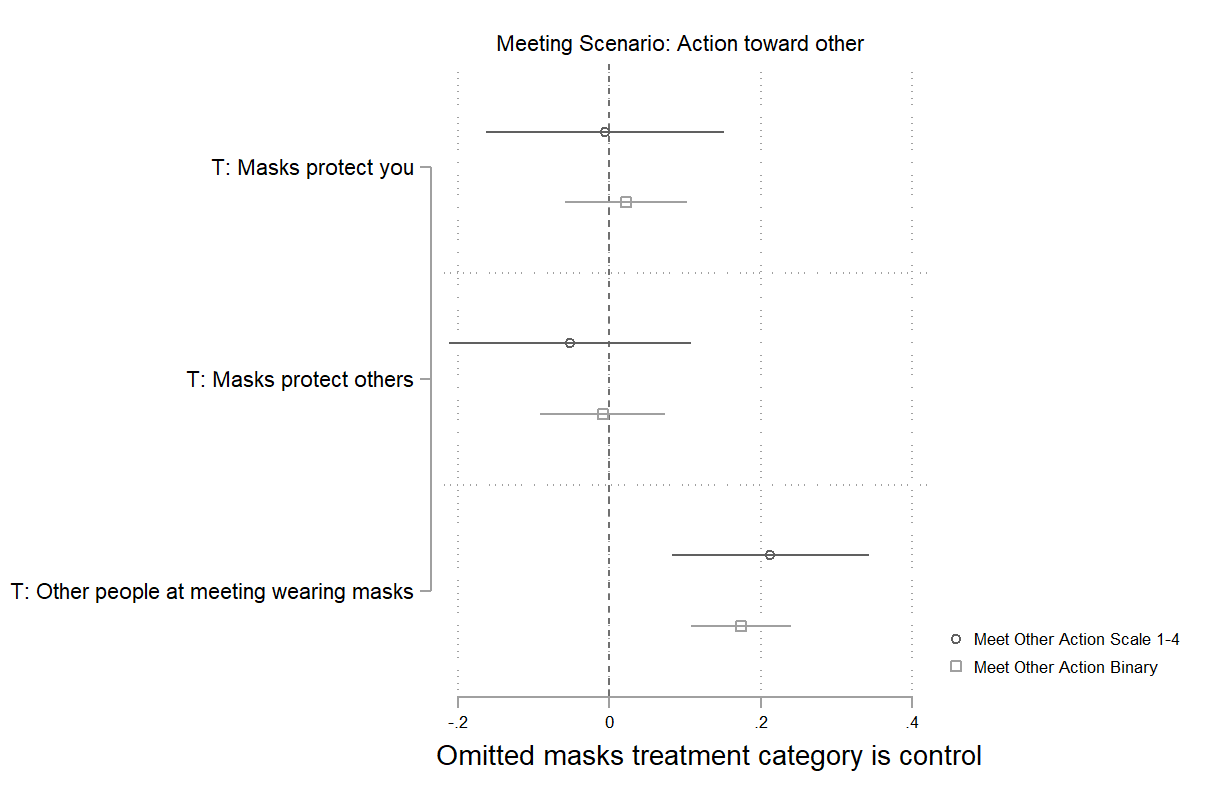** |

Effect of mask efficacy treatments and social norms treatment on reported action towards OTHERS for the MEETING scenario. The figure displays OLS regression estimates with 95% confidence intervals. Models included covariates described above.

**Figure: THIRD PARTY behavior for MEETING Scenario**

| United States | Italy |
| --- | --- |
| **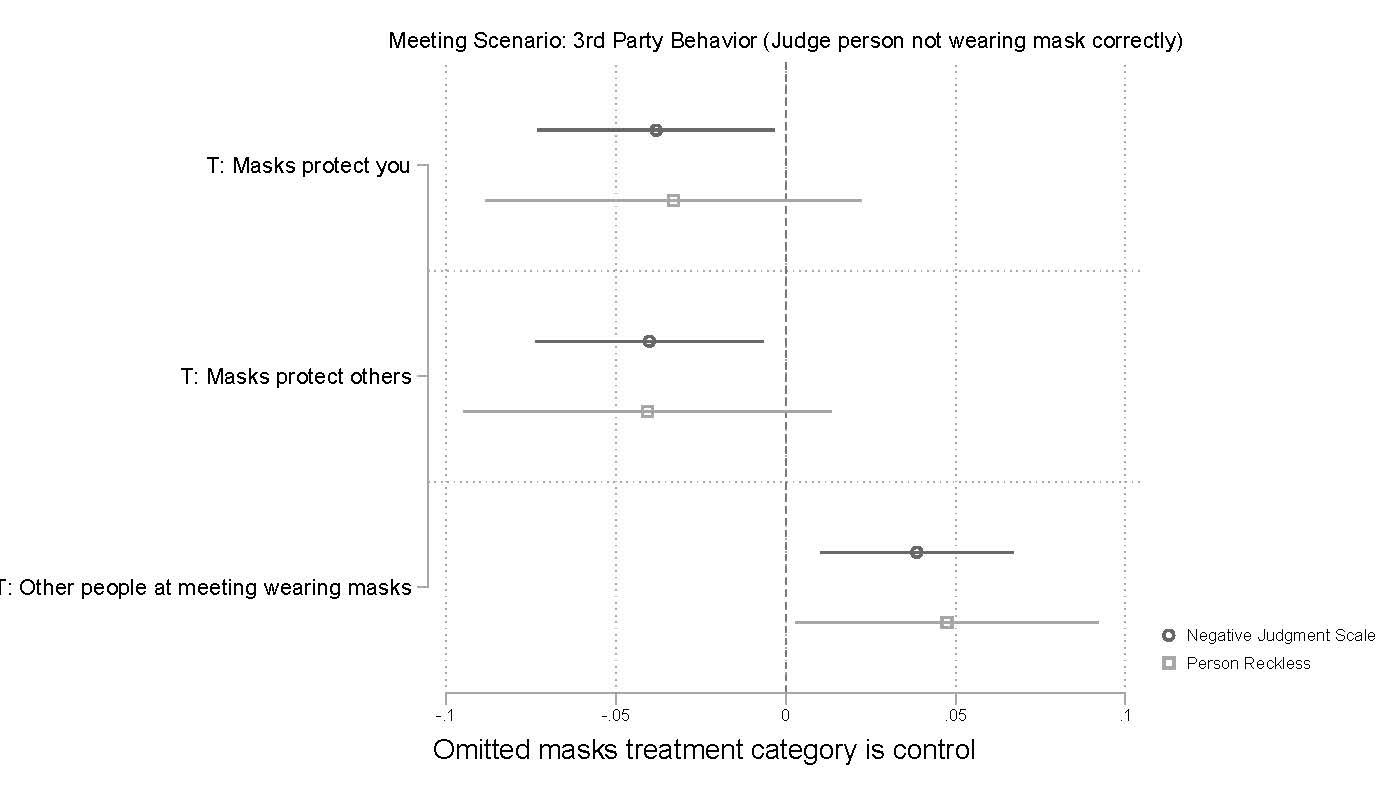** | **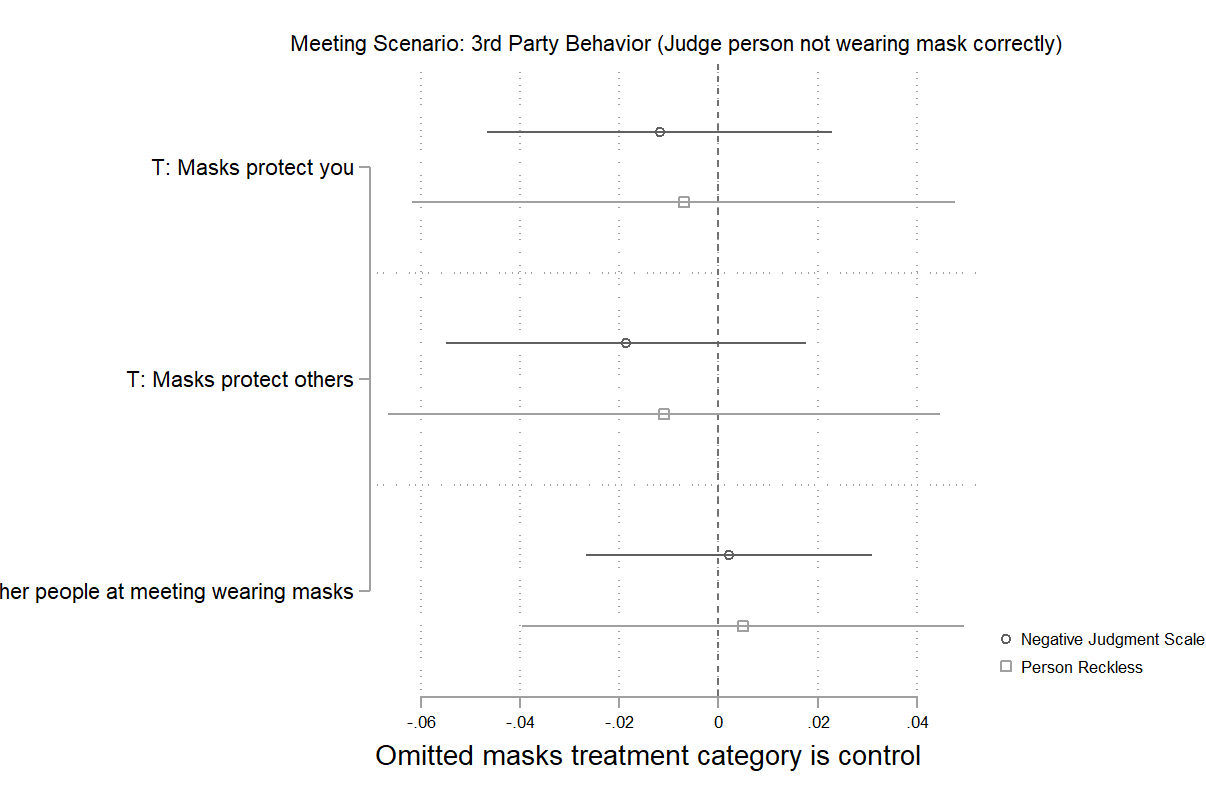** |

Effect of mask efficacy treatments and social norms treatment on reported judgment of person who is not wearing their mask correctly mask behavior for the THIRD PARTY version of MEETING scenario. The figure displays OLS regression estimates with 95% confidence intervals. Models included covariates described above.

**Figure: THIRD PARTY behavior for MEETING Scenario ask to fix**

| United States | Italy |
| --- | --- |
| **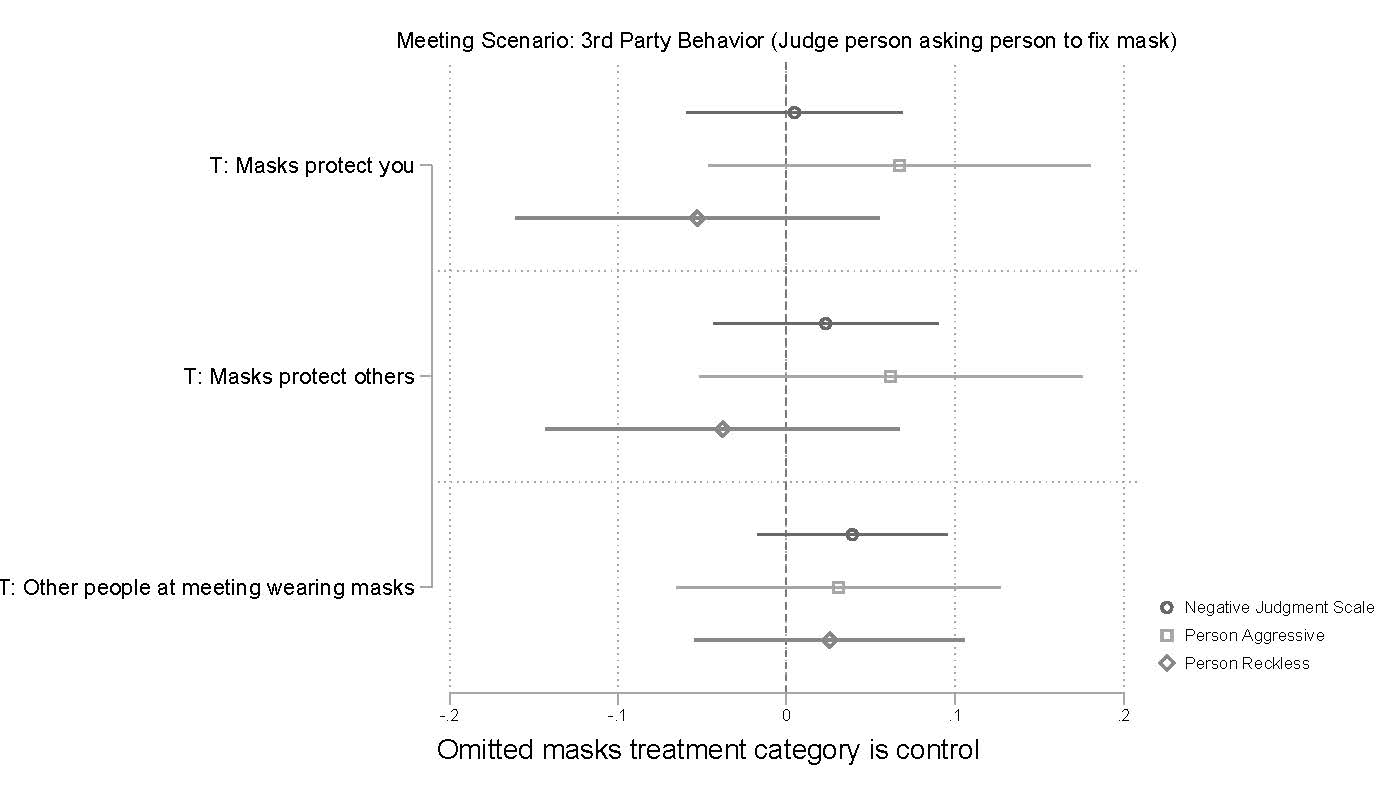** | **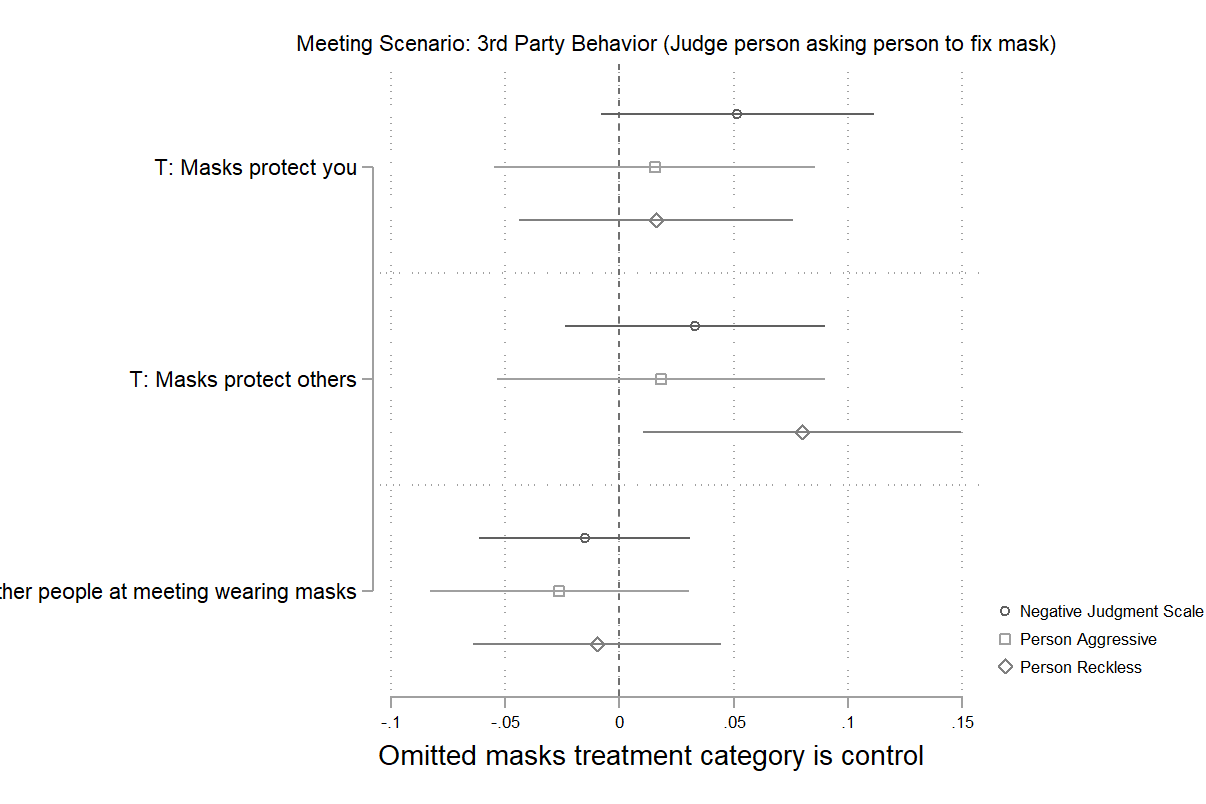** |

Effect of mask efficacy treatments and social norms treatment on reported judgment of person who asked someone to fix their mask in the THIRD PARTY version of MEETING scenario. The figure displays OLS regression estimates with 95% confidence intervals. Models included covariates described above.
