# Supplementary material for: Experimental evidence that changing beliefs about mask efficacy and social norms increase mask wearing for COVID-19 risk reduction: Results from the United States and Italy
Source: PLoS One. 2021 Oct 11;16(10):e0258282. doi: 10.1371/journal.pone.0258282 (PMC8504748; doi:10.1371/journal.pone.0258282)
Supplement: S1 Table — (DOCX) [file pone.0258282.s006.docx]

S1 Table Demographic Information

|  | United States | |  | Italy | |
| --- | --- | --- | --- | --- | --- |
|  | Mean | SD |  | Mean | SD |
| Age | 45.2 | 17.3 |  | 46.8 | 15 |
| Proportion Female | 0.482 | 0.5 |  | 0.526 | 0.5 |
| Proportion Democrat | 0.465 | 0.499 |  |  |  |
| Proportion Republican | 0.389 | 0.487 |  |  |  |
| Proportion Left coalition |  |  |  | 0.177 | 0.381 |
| Proportion Right coalition |  |  |  | 0.255 | 0.436 |
| Proportion 5 Star Movement |  |  |  | 0.247 | 0.431 |
| Proportion College Education | 0.433 | 0.496 |  | 0.218 | 0.413 |
| Proportion White | 0.725 | 0.447 |  |  |  |
| Proportion Black | 0.125 | 0.330 |  |  |  |
| Proportion Northeast United States | 0.203 | 0.402 |  |  |  |
| Proportion Midwest United States | 0.188 | 0.391 |  |  |  |
| Proportion Southern United States | 0.377 | 0.485 |  |  |  |
| Proportion Northeast Italy |  |  |  | 0.189 | 0.392 |
| Proportion Northwest Italy |  |  |  | 0.269 | 0.443 |
| Proportion Center Italy |  |  |  | 0.2 | 0.4 |
| Proportion South Italy |  |  |  | 0.243 | 0.429 |
| Proportion Islands Italy |  |  |  | 0.099 | 0.298 |
|  |  |  |  |  |  |

Descriptive statistics for United States and Italy experiments.
